# “As an ethnic minority, you just have to work twice as hard.” Experiences and motivation of ethnic minority students in medical education

**DOI:** 10.1007/s40037-021-00679-4

**Published:** 2021-09-13

**Authors:** Ulviye Isik, Anouk Wouters, Petra Verdonk, Gerda Croiset, Rashmi A. Kusurkar

**Affiliations:** 1 Amsterdam UMC, Research in Education, Faculty of Medicine, Vrije Universiteit, Amsterdam, The Netherlands; 2 LEARN! Research Institute for Learning and Education, Faculty of Psychology and Education, VU University Amsterdam, Amsterdam, The Netherlands; 3 Amsterdam UMC, Department of Ethics, Law & Humanities, Amsterdam Public Health Research Institute, Amsterdam, The Netherlands; 4 University Medical Center Groningen, Groningen, The Netherlands

**Keywords:** Ethnicity, Intersectionality, Medical students, Motivation

## Abstract

**Introduction:**

Adequate representation of ethnic minority groups in the medical workforce is crucial for ensuring equitable healthcare to diverse patient groups. This requires recruiting ethnic minority medical students and taking measures that enable them to complete their medical studies successfully. Grounded in self-determination theory and intersectionality, this paper explores the experiences of ethnic minority medical students across intersections with gender and other categories of difference and how these relate to students’ motivation.

**Methods:**

An explorative, qualitative study was designed. Six focus groups were conducted with 26 ethnic minority students between December 2016 and May 2017. Thematic analysis was performed to identify, analyse and report themes within the data.

**Results:**

The findings were categorized into three main themes: the role of autonomy in the formation of motivation, including students’ own study choice and the role of their family; interactions/‘othering’ in the learning environment, including feelings of not belonging; and intersection of ethnic minority background and gender with being ‘the other’, based on ethnicity.

**Discussion:**

Ethnic minority students generally do not have a prior medical network and need role models to whom they can relate. Ensuring or even appointing more ethnic minority role models throughout the medical educational continuum—for example, specialists from ethnic minorities in teaching and/or mentoring roles in the education—and making them more visible to students is recommended. Moreover, a culture needs to be created in the educational environment in which students and staff can discuss their ethnicity-related differences.

**Supplementary Information:**

The online version of this article (10.1007/s40037-021-00679-4) contains supplementary material, which is available to authorized users.

## Introduction

To provide equitable healthcare for an increasingly diverse population, a culturally competent healthcare workforce in which ethnic minority groups are adequately represented is a necessity [1]. Thus, breaking down systemic barriers that can prevent ethnic minority students from pursuing medical careers is critical. This requires not only recruiting medical students from ethnic minorities, but also ensuring successful completion of their studies. Often, medical students from ethnic minorities underperform compared to the majority group [2, 3] and have difficulty procuring placements for post-graduate medical education [4]. Motivation is often used to explain lower performance [5], but it can also explain how difficulties, barriers and experiences influence ethnic minority students’ motivation. However, ethnic minority students’ unique experiences in the medical curriculum and how these relate to their motivation have not been investigated. Such knowledge may inform interventions to help these students complete their studies successfully.

We used the self-determination theory’s (SDT) concept of motivation as our framework [6, 7]. SDT distinguishes between autonomous and controlled motivation types. Autonomous motivation arises from genuine interest or finding an activity personally important. Controlled motivation arises from internal or external pressure or desire for a reward. SDT also describes a hierarchical model of motivation with global, contextual and situational levels [8]. The global level concerns a global motivational orientation within which the individual interacts with their environment [8–10]. Contextual motivation relates to the individual’s life context, like education or work and in this study, a student’s motivation for medical study and practice. Situational motivation pertains to a particular time, place and activity, and it can be influenced by social factors, such as a student’s interaction with a specialist during a clerkship. These levels affect each other reciprocally [8]. Taking part in autonomously motivating activities at the situational level facilitates contextual autonomous motivation.

Motivation is dynamic and can be influenced by environmental factors, like stereotype threat [6, 11]. Three basic psychological needs must be satisfied for autonomous motivation: autonomy (feeling of choice), competence (feeling capable of mastering challenges), and relatedness (feeling connected to others). Satisfaction of these needs can transform controlled motivation for that activity into autonomous motivation [12]. Four main factors influence ethnic minority students’ motivation both positively and negatively: individual factors (e.g., learning-related emotions), family-related factors (e.g., family support), school-related factors (e.g., teacher support), and social factors (e.g., discrimination) [13]. These factors suggest that experiences related to social identities play a role in motivation.

Intersectionality refers to the interactions between gender, race, religion and other categories of difference in individual lives, social practices, institutional arrangements and cultural ideologies, and the outcomes of these interactions in terms of power [14, 15]. It posits that people are located on axes of difference across these categories, which creates unique positions that provide disadvantage and privilege based on historical group relations. People who are perceived to belong to a group that does not live up to dominant norms are created as ‘Other’, and their experiences are considered less legitimate and credible [16]. In this study, we adopted an intersectional approach to ensure that ‘ethnic minorities’ are not treated as a homogeneous group with no within-group differences [14, 17, 18]. Exploring the interactions between all these aspects enables a deeper understanding of ethnic minority students’ experiences. Thus, in this study, we investigate how medical students’ ethnic identities and their intersection with other aspects of diversity relate to their motivation.

## Methods

We conducted an explorative, qualitative study in which we adopted an intersectional approach to study the interactions between gender, race and other categories of difference in individual lives of ethnic minority students. We adopted a constructivist paradigm in which knowledge is constructed through interaction between the researchers and participants [19, 20]. The Ethical Review Board of the Netherlands Association for Medical Education granted ethical approval (NVMO, file #663). All participants provided informed consent. The Consolidated Criteria for Reporting Qualitative Research checklist and Standards for Reporting Qualitative Research guided our reporting [21, 22].

Ethnic minority medical students enrolled at the Faculty of Medicine VU Amsterdam, the Netherlands, were invited per email by the first author (UI) to participate. Our school enrolls approximately 30% ethnic minority students and has a 3-year Bachelor (with preclinical components) and 3‑year Master programme (with clinical components) [23].

We used the definition of ethnic minority from Statistics Netherlands [2, 4]. According to it [24], the population of the Netherlands is comprised of a 77% Dutch majority, a 13% non-Western minority (e.g., Turkish, Moroccan, Surinamese) and a 10% Western minority (e.g., Indonesian, German, Polish). Although the number of ethnic minority students has increased substantially, only 2%–4% of the medical specialists have an ethnic minority background [2, 25]. To determine participants’ ethnic minority status, we questioned students about their birth country and that of their parents.

Purposive sampling was used to ensure representation from different ethnic groups and to gather rich and suitable data [26]. We also used snowball sampling by asking the participants to recommend other students to participate [27]. Separate focus groups were held with Bachelor and Master students because of their different learning environments: the Bachelor programme is more campus-based and the Master programme practice-based.

Using focus groups, we aimed to achieve diversity in participants’ profiles leading to rich responses and perspectives [28]. We anticipated that the group setting would stimulate participants to describe their experiences and to relate each other’s comments to their own lived experiences. Focus groups were conducted from December 2016–May 2017. UI, who has an ethnic minority background, moderated the focus groups using a semi-structured interview format (see the Appendix included in the Electronic Supplementary Material [ESM]) informed by SDT and intersectionality. A female Master ethnic minority medical student checked the interview guide to ensure it included a student’s perspective. A research assistant (BvE) took field notes and observed the groups’ interactions. After the focus groups, UI and BVE debriefed the experience. Focus groups ranged from 71–151 min and were conducted until we could adequately answer the study’s research question. Focus group discussions were audiotaped, transcribed verbatim and de-identified. To ensure accuracy, quotes were translated to English by a native Dutch speaker, checked by a native Dutch speaker fluent in English and then edited by a native English speaker fluent in Dutch.

We applied thematic analysis for identifying, analysing and reporting patterns (themes) within our data [29] using SDT as the theoretical framework [6, 7]. In addition, an intersectional approach was adopted to cluster the data into themes [14, 17, 18]. UI coded and managed all data in Microsoft Excel. Two other two researchers (AW and PV) each coded two transcripts independently. In addition, PV read all transcripts and made memos based on her diversity expertise. The research team discussed all findings until consensus was reached.

The research team’s diversity helped us to stay critical, gain depth and create dialogue about the findings from different perspectives. UI (a female PhD student) and RAK (a female senior researcher in motivation) have ethnic minority backgrounds. AW (a female post-doc researcher), PV (a female senior researcher in intersectionality) and GC (a female senior researcher and pioneer of diversity in the university) have ethnic majority backgrounds. Research assistant (BvE) is male with an ethnic majority background.

## Results

We conducted six focus groups in total: three with bachelor (*n* = 15) and three with master students (*n* = 18). Twenty-six ethnic minority students participated (*n* = 18 female). Fourteen participants were first-generation university students. Parents’ countries of birth included two Western countries (Spain and Indonesia) and twelve non-Western ones: Afghanistan, China, Curacao, Egypt, Ghana, Kuwait, Morocco, Russia, Sudan, Suriname, Turkey and Vietnam.

We categorized our findings into three themes: the role of autonomy in the formation of motivation, interactions and othering in the learning environment, and the intersection of minority background and gender. To provide context, we have incorporated participant quotes that include notation of their characteristics, including participant number, sex, and programme (e.g., S2, M, preclinical).

### The role of autonomy in the formation of motivation

Students expressed feelings about autonomy and motivation, such as their own study choice, and how their family and culture played a role. They explored the importance of their family in developing their own autonomous motivation for studying medicine in several ways, e.g., family support, family expectations, proud parents, and being or having a role model in the family. The following student seems to have successfully integrated the norm of conforming to group expectations (i.e., his family’s expectations of him becoming a doctor) with the norm of making one’s own autonomous choice:* “Uh, I was actually expected to … I have a lot of doctors in my family, so they expected me to do that too. But in the end, I was pretty resistant to it, I just looked at what I found most interesting and, by chance, that was also medicine” *(S2, M, preclinical).

### Interactions and othering in the learning environment

Within the theme of interactions and othering in the learning environment, we identified several subthemes.

Othering/not belonging: Students discussed interactions in practice, in which ‘othering’ and hierarchy both played roles. A participant expressed how they (ethnic minority students) did not fit into the student team or the department team because of cultural differences that led to feelings of ‘not belonging’. According to the student, exclusion works as follows:* “[…] they are looking for someone who fits in their team. And if the team is made up of people who like to go skiing and drink beer, and you come in with your headscarf and you do not drink alcohol, you do not go skiing and you pray five times a day, then they say: ‘No, we do not want her’” *(S3, M, clinical).

Another student’s story illustrates that students anticipate demotivating experiences based on their cultural background, affecting their sense of belonging: “I think that it is not different once you are a resident, then you just look for having a connection. Then you have a group of residents that you relate better with. Yes, and unfortunately, that often leads to segregation into non-Western and Western physicians and students. If I walk around in a hospital and I see only blond Dutch people, that is demotivating for me.”Ni-vo-lu-mab-grup-pe (S3, M, clinical).

Students mentioned various factors and expectations related to their study and cultural background that negatively influenced their motivation, in particular needing to stand up for oneself and against being excluded as ‘the other’. A student had an experience in which someone described her as ‘an exception to the rule’ in comparison to other ethnic minority students. As a consequence, ethnic or cultural background is framed as ‘less than’, ‘not as competent as’. Such an ‘othering’ experience communicates to ethnic minority students that ‘the rule’, the expectation, is that ethnic minority students do not speak Dutch fluently, or that they underperform in medical school, and is experienced as an insult wrapped in a compliment.

Lack of role models: Another student indicated that a lack of role models (from ethnic minorities) could negatively influence students’ motivation to become specialists: “Yes, you do not often see a specialist who has an ethnic minority background. Um, so now and then I sometimes wonder: ‘Why should I study so hard for six years?’ In the end, I probably cannot do what I want to do. Or, yes, I do not want to become a specialist, but rather a GP or something else, a Medical Doctor or … Because you do not see that you can get there, that can be very demotivating. If you look at what the flow of medical students from ethnic minorities is that will eventually get into speciality training, that is extremely low compared to the entry of medical students.” (S12, M, preclinical).

Communication difficulties: Students mentioned the Dutch ‘talking culture’ as problematic, because many ethnic minority students do not share this culture. For example, a student remarked: *“White students are more articulate, or something. We did not learn that at home” *(S12, male, preclinical). Other students mentioned cultural differences in their understanding of assertiveness by comparing the Dutch majority culture with their ethnic minority cultures:* “I received a Fail for my current clerkship. Your cultural background influences your interaction with GPs. It is because everything is hierarchical for us, we are not used to giving a reply … [But] it is seen as not assertive if you do not enter into discussion with a doctor” *(S2, M, preclinical).

Working hard: *“When I was in the bachelor, I got in touch with an ethnic minority physician in specialty training, and he told me that as an ethnic minority you just have to work twice as hard, that’s the way it is”* (S3, M, clinical). This student explained how students from ethnic minorities warn each other; tell each other that the bar is set very high for them. However, despite the struggles of the ethnic minorities, this student and several others stressed that he does not let such things negatively influence his motivation, he just works harder. However, he expects that such experiences could negatively influence other students’ motivation.

Coping mechanisms/creating awareness: Another student made an impression of resilience by giving a positive twist to experiences that were initially influencing her motivation negatively. She stressed that she would not let them demotivate her, and rather, that she had become even stronger.* “Because I am from an ethnic minority, I just grew up like that. But those things cannot really have a negative influence on me because I’m really like, ‘I just can do it’” *(S5, F, preclinical). Another bachelor student illustrated that some students are more resilient than others. More students talked about negative experiences related to their headscarves that did not affect their motivation, but rather made them more aware of the others’ mindset: *“Then I did my clerkship with a GP, and he said: ’Yes, you are, how you participate is all fine, but I am not so happy with that headscarf and it is a pity, and maybe you should take it off’ […]. And then that whole assessment interview was about my headscarf”* (S7, F, clinical).

### Intersection of minority background and gender

Students addressed cultural differences many times. Coming from an ethnic minority background was seen as an advantage and disadvantage: “[…] And by that I am not just referring to patients who look like me, so, for example, um … with Muslims, it does not matter what their background is. I think this kind of empathy that I have, is also for homosexuals I can also find more empathy for them, or for a transgender person. It does not matter a lot from which group they come. Buddhists, Hindus […] I can place myself in the other’s situation faster.” (S8, F, clinical).

This student described how her own experiences help her understand exclusion and its consequences, and how this disadvantage can be turned into an advantage because she learned to feel empathy for people unlike her.

Some students anticipate disadvantages based on being a female with an ethnic minority background:* “No matter how many female medical students there are, once it comes to specialisation, there are simply fewer women. And even fewer of non-Dutch origin. So that’s two things already against us” *(S14, F, clinical).

## Discussion

This study highlights the role of autonomy in the formation of motivation, interactions and othering in the learning environment, and the intersection of minority background and gender, such as being made to feel like ‘the other’ on the basis of both ethnicity and gender. Further, we apply the lens of what SDT describes as global, situational and contextual motivation.

Our findings suggest that culture-related experiences and factors can greatly influence students’ autonomy and relatedness, two of the basic psychological needs in SDT [7, 9]. We illustrated how students’ motivation was integrated with the motivation coming from family expectations. On the one hand, they want to fulfil family expectations; on the other, they desire to make their own decisions. A possible explanation for this paradox of autonomy could be that students from ethnic minorities grow up with two cultures, their own minority collectivistic culture, and the majority individualistic culture. Family relations are crucial in ethnic minority groups with a collectivistic culture [30].

The fulfilment of the basic need for ‘relatedness’ was critical in the students’ experiences and regularly emerged from their stories. A factor that negatively affected their feelings of relatedness and situational motivation was that the hospital staff was largely ethnic majority, which was interpreted as being unrepresentative of the population. Another study among residents of an ethnic minority also indicated similar frustration [4]. Because the residents felt less related and less able to connect with their majority group colleagues, they found it difficult to successfully network [4]. These minorities explained that department heads and senior medical specialists could, and should, play a bigger role in creating a safe environment.

Participants often reported feeling that they are ‘othered’, and hence, excluded, which affects their situational motivation. Fitting into student or department teams seems harder because of their cultural background, (e.g., when they do not engage in typical activities for the majority or because they wear a headscarf) [4, 31]. Research has suggested that such experiences of cultural differences may create difficulties for ethnic minority students (and physicians) in presenting confidently [4, 32–34]. Moreover, these experiences and interactions, remarks about being a Muslim, or wearing headscarves and assertive approaches, are a process of growing awareness for them. The impressions and remarks about appearances could influence assessments and performance grades directly or indirectly. However, it is remarkable that the study participants seldom expressed feelings of anger in these situations. Remaining silent about their insecurities and emotions because that is seen as ‘being professional’ may place ethnic minority students at a disadvantage as compared to the ethnic majority students [35]. Further, being seen as ‘an exception to the rule’ illustrates tokenism: being more visible than others in the group because of some aspect of your appearance [36]. This increased visibility could lead to experiencing pressure to perform. Remarks about being exceptional can also lead to a disconnection from others, where the individual feels excluded from the very group with which they identify. Such disconnection might lead ethnic minority students to disenroll from their studies [34].

The lack of ethnic role models influences the students’ situational motivation and career choices (contextual motivation). This aligns with previous research [37]. Especially for those students who lack a medical network, there seems to be a need for role models when applying to study medicine. Medical students who are first-generation university students also experience difficulties because of the lack of a medical network [38].

Students expressed that remarks about their accents were irritating because they spoke the majority language perfectly. A study performed among ethnic minority residents in the Netherlands showed that the difference did not involve language skills but rather the different types of language used and the way language is used socially [4]. In addition, students experienced difficulties related to the reactions they received about their supposed lack of assertiveness. This can disadvantage ethnic minority students when functioning in a majority society that encourages assertiveness [39]. This raises the questions for future research: How is assertiveness expressed and valued by students and doctors from different ethnic minorities as opposed to people from the majority culture? What deserves attention is the match between the medical community’s idea of assertiveness in patient care and patients’ expectation of assertiveness from the doctors.

Students expressed how cultural background could form a barrier to their display of expected behaviour, for instance when relating in a hierarchical relationship with a doctor, and adhering to norms of being respectful, influencing their situational motivation. Students find it challenging to proactively ask their clinical supervisor questions because it is considered disrespectful in their culture. This places minority students in an even more disadvantaged position in hierarchical relationships than majority students, and students may adopt the expected attitude while still having difficulty integrating it, which can lead to additional stress.

Using intersectionality in this study exposed power differentials and hierarchies between ethnic groups; it exposed how individuals are members of multiple groups but also have unique identities defined by their social positions [4, 14, 32]. A few students expressed negative experiences related to being female and having an ethnic minority background, which causes a sense of being ‘othered’. These women run the risk of eventually learning to devalue themselves as medical students and future physicians, which undermines their professional development [31]. Students’ narratives showed that their experiences at a particular moment could negatively influence their (situational) motivation, but in the long term, they learned to cope with it. Thus, they retained motivation for their medical studies and their ultimate goal of becoming a doctor (contextual motivation). This shows that situational motivation—in this case, towards a goal—is mainly influenced by the contextual motivation for a goal [8]. Being unable to cope with recurrent, negatively influencing situational experiences may demotivate students over time, and thus, negatively affect their motivation to continue their medical studies (contextual motivation). See Tab. 1 for practical suggestions resulting from our findings.

**Table 1:** Suggestions for creating a better learning environment for ethnic minority students

Subtheme Addressed	Suggestion
Othering/not belonging	Create an environment in the learning environment where ethnic minority students can feel that they have a place. For example providing (small) support groups for students with different ethnic backgrounds to share experiences and support each other
Othering/not belonging	Create a ‘low threshold’ system for reporting discrimination and insults if desired
Lack of role models	Ensure better representation of role models, for example specialists, with an ethnic minority background in teaching, tutoring and mentoring roles across the educational continuum
Communication difficulties	Provide clarity about what is expected of students by considering ethnic minority students’ cultural backgrounds, and guiding students (using role models) regarding the expectations such as assertiveness in a sensitive manner
– Communication difficulties – Coping mechanisms/creating awareness	Educate staff and students about the culture of medicine, and how to communicate about ethnic differences to create cultural awareness and competence
– Working hard – Influence of hierarchy – Othering/not belonging – Coping mechanisms/creating awareness	Discuss ethnic and cultural differences in the learning environment

## Limitations

Given the researchers’ ethnic minority backgrounds and familiarity with the experiences, certain topics may have remained underexplored in the focus groups and analysis [31]. Another limitation is the limited transferability of the findings as each country has its own unique ethnic minority subgroups and unique distributions of these subgroups. We cannot assume that all students with a minority background have similar experiences, although being a minority may result in shared understandings. Thus, these findings are ‘credible’ in that they resonate with other research across countries [13], and these findings may well apply to other settings. By using the ‘ethnic minority’ definition of Statistics Netherlands, we may have excluded potential participants who identify as ethnic minority but are excluded by the definition. Future researchers should consider using Ross et al.’s definition of ethnicity, which allows participants to define their preferred ethnic background [40]. Furthermore, by using snowball sampling, we may have attracted more resilient or motivated ethnic minority students to participate. It is possible that students who are less resilient and motivated have different experiences.

## Conclusion

Applying an SDT lens to our findings helped us to understand how the students’ backgrounds and experiences formed and affected their motivation for their medical studies. Moreover, it gave us insight into how students’ experiences can influence their academic motivation in the short term (situational motivation) as well as in the long term (contextual motivation). Our study shows that students’ experiences at particular moments could influence their situational motivation related to particular activities in their medical studies negatively. However, in the longer term, participants learned to cope with this and sustained their contextual autonomous motivation through sustained genuine interest or personal endorsement of the importance of their medical studies. Nevertheless, the negative experiences may keep them from performing to their full potential.

## Supplementary Information

The interview guide.
