# Supplementary material for: “As an ethnic minority, you just have to work twice as hard.” Experiences and motivation of ethnic minority students in medical education
Source: Perspect Med Educ. 2021 Sep 13;10(5):272–8. doi: 10.1007/s40037-021-00679-4 (PMC8505584; doi:10.1007/s40037-021-00679-4)
Supplement: Supplementary file 1 — The interview guide. [file 40037_2021_679_MOESM1_ESM.docx]

**Appendix** Interview guide

|  | What is your (main) reason for studying medicine? |
| --- | --- |
|  | What do you need to be motivated/ what motivates you/ what stimulates your motivation? |
|  | What kind of influence do the experiences in the learning environment (during the courses, and contacts with peers, teachers, and non-teaching staff) have on your motivation? Can you give examples? |
|  | Which experiences in the learning environment have/had a positive influence on your motivation? |
|  | Which experiences in the learning environment have/had a negative influence on your motivation? |
|  | Do you also have (positive or negative) experiences that are related to your culture? Or your ethnicity? |
|  | Did these experiences influence how competent you feel for this study? How did you deal with this? |
|  | When did you have the feeling that you were connected with your peers, teachers, and in general to this study? And what kind of influence did this have on your motivation, study, studying, to become a doctor? |
|  | What does autonomy mean for you? Which messages about autonomy have you learnt at home? Do you think that this is culturally determined? And how? |
|  | When do you feel autonomous? (depending on what participants mean by autonomy) Do you ever feel less autonomous? Which aspects/experiences in the learning environment have influenced this? |
|  | What influence does your motivation have on your academic performance? |
|  | Are there other important aspects (related to social and cultural factors that influenced your motivation) that you want to bring to the attention of the researchers? |
